# Angiopoietin-1 promotes triple-negative breast cancer cell proliferation by upregulating carboxypeptidase A4: ANG1-CPA4 axis promotes TNBC progression

**DOI:** 10.3724/abbs.2023082

**Published:** 2023-05-09

**Authors:** Xue Liu, Huichun Liang, Huan Fang, Ji Xiao, Chuanyu Yang, Zhongmei Zhou, Jing Feng, Ceshi Chen

**Affiliations:** 1 Medical College Anhui University of Science and Technology Huainan 232001 China; 2 Department of Laboratory Medicine & Central Laboratory Fengxian District Central Hospital of Shanghai Shanghai 201499 China; 3 Key Laboratory of Animal Models and Human Disease Mechanisms of the Chinese Academy of Sciences and Yunnan Province Kunming Institute of Zoology Chinese Academy of Sciences Kunming 650201 China; 4 College of Life Science and Technology Guangzhou Jinan Biomedicine Research and Development Center Jinan University Guangzhou 510632 China; 5 The School of Continuing Education Kunming Medical University Kunming 650500 China; 6 Academy of Biomedical Engineering Kunming Medical University Kunming 650500 China; 7 The Third Affiliated Hospital Kunming Medical University Kunming 650106 China; 8 The Second Affiliated Hospital of the Chinese University of Hong Kong Shenzhen 518172 China

**Keywords:** angiopoietin-1, carboxypeptidase A4, cell proliferation, cell cycle, triple-negative breast cancer

## Abstract

Angiopoietin-1 (ANG1) is a pro-angiogenic regulator that contributes to the progression of solid tumors by stimulating the proliferation, migration and tube formation of vascular endothelial cells, as well as the renewal and stability of blood vessels. However, the functions and mechanisms of ANG1 in triple-negative breast cancer (TNBC) are unclear. The clinical sample database shows that a higher level of ANG1 in TNBC is associated with poor prognosis compared to non-TNBC. In addition, knockdown of *ANG1* inhibits TNBC cell proliferation and induces cell cycle G1 phase arrest and apoptosis. Overexpression of ANG1 promotes tumor growth in nude mice. Mechanistically, ANG1 promotes TNBC by upregulating carboxypeptidase A4 (CPA4) expression. Overall, the ANG1-CPA4 axis can be a therapeutic target for TNBC.

## Introduction

Among women, breast cancer is the most frequently occurring cancer, accounting for approximately 11.7% of female cancer cases and the primary cause of cancer deaths in 2020 [1]. Breast cancer is subdivided into four subtypes based on estrogen receptor (ER), progesterone receptor (PR) and human epidermal growth factor-2 (HER2) expression, of which the subtype that is all receptor negative is triple-negative breast cancer (TNBC) [2]. TNBC has a high recurrence and metastasis rate and is heterogeneous [3]. Despite advances in clarifying tumor biology, effective treatment therapies for TNBC are unfortunately still limited due to its intense resistance to radiotherapy and chemotherapy [ 4 – 7]. Thus, there is an urgency to clarify the potential mechanisms of TNBC progression to identify novel and effective targets for TNBC treatment.

Angiopoietin-1 (ANG1), a member of the angiopoietin family, is normally secreted by perivascular cells and binds to tyrosine kinase receptor 2 (Tie2) on endothelial cells, which plays an important role in vascular integrity, vessel remodeling, vessel enlargement, and anti-inflammation [8]. The interaction between ANG1 and Tie2 promotes the survival of endothelial cells by activating the PI3K/AKT pathway [9], regulates the endothelial cytoskeleton by activating the IQGAP1/Rac1 pathway [10], and enhances the anti-inflammation of the vasculature by inhibiting the NF-κB pathway [11]. In addition, ANG1 is secreted by tumor cells and activates Tie2 on endothelial cells, which increases vascular perfusion in tumors by promoting angiogenesis and enlarging the vascular lumen [ 12– 14]. Our previous work also confirmed that ANG1 secreted by breast cancer cells promotes the migration and tube formation of endothelial cells [15]. However, the functions and mechanisms of ANG1 in breast cancer cells are unclear and are valuable for us to investigate.

Carboxypeptidase A4 (CPA4) belongs to the zinc-containing metallocarboxypeptidase family, which is catalytic for the release of carboxy terminal amino acids and is principally used for the cleavage of C-terminal fragments of both proteins and peptides [16] and may also be responsible for the regulation of peptide hormone activation and hormonal modulation of tissue development and differentiation [ 17– 19]. CPA4 is a novel biomarker for cancer that is prevalent in a variety of cancers and is recognized to regulate inflammation, playing an essential role in the tumor microenvironment as well as tumor cell proliferation and invasion [ 17, 20– 22]. Elevated serum level of CPA4 has been reported to be associated with disease invasion and metastasis [23], poor prognosis [ 18, 20, 24, 25], and progression in various tumors and has some referential value in diagnosis and prognosis [ 21 , 26– 29]. CPA4 has also been reported to promote stemness [ 30 , 31] and epithelial mesenchymal transition [17] in different types of cancers.

In this work, we revealed that ANG1 promotes the proliferation of TNBC cells by upregulating the CPA4/Cyclin D1 axis. Deficiency of ANG1 was observed to induce apoptosis and cell cycle G1 phase arrest. Our results may provide new insight into the mechanism by which ANG1 enhances the development of cancer and indicate that the ANG1/CPA4 axis has the potential to be a novel target for TNBC treatment.

## Materials and Methods

### Bioinofmatics analysis

The expression of *ANG1* and *CPA4* genes in TNBC was analyzed by using bc-GenExMiner v4.9 ( http://bcgenex.ico.unicancer.fr/), a statistical mining tool of published annotated breast cancer transcriptomic data. It offers the possibility to explore gene-expression of interest in breast cancer. Overall survival analysis of breast cancer patients with high ANG1 or CPA4 expression using the breast cancer integrative platform ( www.omicsnet.org/bcancer).

### Cell lines and culture

The cell lines used in this study were purchased from the American Type Culture Collection (ATCC; Manassas, USA) and authenticated by the STR typing assay. Hs578T, MDA-MB-468 and HEK293T cell lines were cultured in DMEM (Gibco, Carlsbad, USA) supplemented with 5% fetal bovine serum (FBS; Gibco). HCC1806 and HCC1937 cell lines were maintained in RPMI 1640 medium (Gibco) with 5% FBS. SUM149PT cells were grown in Ham’s F12 medium (Gibco) supplemented with 10% FBS, 5 μg/mL insulin and 1 μg/mL hydrocortisone. The other cell lines used in this study were cultured according to the ATCC suggestions. These cells were cultured at 37°C with 5% CO _2_.

### Cell transfection

Hs578T and SUM149PT cells were seeded in 6-well plates at densities of 2.0×10 ^5^ and 2.5×10 ^5^ cells per well, respectively. On the next day, Lipofectamine 2000 (Invitrogen, Carlsbad, USA) was used for siRNA (Tsingke Biotechnology, Beijing, China) transfection at a final concentration of 50 nM according to the manufacturer’s suggested protocols. The siRNA target sequences for the human *ANG1* gene were 5′-GGAAGAGAAAGAGAACCTT-3′ (siANG1-1) and 5′-CTATGATGATTCGACCTTT-3′ (siANG1-2). The siRNA target sequences of the human *CPA4* gene were 5′-GATGATGAAATGCAACACA-3′ (siCPA4-1) and 5′-CTGGAACGCTAGTTTTGCA-3′ (siCPA4-2). The Cat. number of control siRNA was TSGXS101 (Tsingke Biotechnology).

### Western blot analysis

The protease inhibitor P8340 (Sigma, St Louis, USA) was added to RIPA lysis buffer at a ratio of 1:100. The cells were treated with lysis buffer on ice for 35 min. After centrifugation to remove cell debris and quantification, approximately 30 μg of protein was resolved by 9%–10% SDS-PAGE and then transferred onto polyvinylidene fluoride (PVDF) membranes. Then, the membrane was blocked with 5% milk for 1 h at room temperature and incubated with the corresponding primary antibody for more than 10 h at 4°C. Finally, secondary antibodies were incubated at room temperature for one hour before being visualized on a biomolecular imager to determine the differential expression of proteins. The anti-Cyclin D1 antibody (#55506S), anti-CDK4/6 antibodies (#12790S and #3136S), and anti-p21/p27 antibodies (#2947S and #3686S) were all purchased from Cell Signaling Technology (CST; Danvers, USA) and used at a 1:1000 dilution with 3% BSA. The anti-ANG1 and anti-CPA4 antibodies were purchased from Proteintech (#23302-1-AP and #26824-1-AP; Rosemont, USA). The anti-Flag and anti-Vinculin antibodies were purchased from Sigma (#F3165 and #V9131).

### ELISA

After cells were seeded into 12-well plates at 1.2×10 ^5^ cells/well, the supernatant was collected at approximately 72 h when the cell confluence reached 90%, and the conditioned culture was placed on ice in 1.5-mL Eppendorf (EP) tubes. Secretion levels of ANG1 in conditioned medium were quantified after centrifugation at 800 *g* for 5 min at 4°C according to the manufacturer′s recommendations using the Human ANG-1 quantitative ELISA kit (R&D Systems, Shanghai, China).

### Proliferation assay

Cell viability was measured by sulforhodamine B (SRB; Sigma) assay. The cells were plated in 96-well plates at 5–6×10 ^3^ cells/well in a volume of 100 μL culture medium. After cultivation for 24, 48, 72, and 96 h, the cells were treated with 10% trichloroacetic acid (TCA; Sigma) at room temperature for 2 h, followed by incubation with 0.4% SRB solution in 1% acetic acid for 30 min at room temperature. Then, the samples were washed 4 times with acetic acid for air drying. Finally, 10 mM unbuffered Tris-base was used to dissolve SRB, and the absorbance was measured at a single wavelength of 530 nm on an Epoch microplate reader (BioTek, Winooski, USA). For the colony formation assay, the cells were seeded into 12-well plates at a density of 1×10 ^3^ cells/well and then incubated with the corresponding medium for 14 days. Then the number of colonies were counted. All experiments were conducted independently more than three times.

### RNA extraction and quantitative reverse transcription PCR (RT-qPCR)

Total mRNA was extracted using TRIzol® reagent (Invitrogen, Carlsbad, USA), and cDNA was synthesized using reverse transcription kits (Vazyme Biotech, Nanjing, China). The expression levels of target mRNAs were quantified using SYBR PCR Master Mix (Applied Biosystems, Foster City, USA) on an ABI-7900HT fast real-time PCR system (Applied Biosystems). The *ANG1* gene qPCRprimer sequences were as follows: forward, 5′-CGTGGAACCGGATTTCTCTTC-3′ and reverse, 5′-CGAGAAGTTTGATTTAGTACCTGG-3′. The primer sequences of *CPA4* were as follows: forward, 5′-CTGGACGGCAAGGAAGATTGT-3′ and reverse, 5′-GACCGCGTCTTCCTCCATAA-3′. The *18S* primer sequences were as follows: forward, 5′-CTCAACACGGGAAACCTCAC-3′ and reverse, 5′-CGCTCCACCAACTAAGAACG-3′.

### Knockdown and overexpression of *ANG1*

ANG1 and control shRNAs were expressed using pSIH1 lentiviral vectors. The target sequences of ANG1 siRNA and shRNA are consistent, as follows: 1#, 5′-GGAAGAGAAAGAGAACCTT-3′; 2#, 5′-CTATGATGATTCGACCTTT-3′. The ANG1 overexpression plasmid was derived from previous studies [15]. The viral particles were prepared by transfecting HEK293T cells with the constructed plasmids or control plasmids in combination with packaging vectors. Viral supernatant was harvested and passed through 0.45-μm syringe filter at 48 h after transfection. After lentivirus infection, cells were screened using puromycin to obtain a stable cell population.

### Cell cycle analysis

After 48 h of ANG1 siRNA interference, SUM149PT and Hs578T cells were stained with propidium iodide (PI; BD Biosciences, San Diego, USA) and RNase A buffer for 30 min at room temperature, followed by analysis via flow cytometry (BD Biosciences) within 2 h.

### Apoptosis analysis

Hs578T and SUM149PT cells were seeded in 6-well plates at densities of 1.8×10 ^5^ and 2.3×10 ^5^ cells/well, respectively. The next day, the cells were transfected with siRNAs for 48 h. All cells and culture medium were collected and stained with Annexin V and PI (BD Biosciences). Finally, an Accuri C6 Flow Cytometer (BD Biosciences) was used to analyze apoptosis.

### RNA sequencing

Total RNA of Hs578T and SUM149PT cells (1×10 ^8^ cells/sample) with control siRNA or ANG1 siRNA treatment was extracted by using TRIzol reagent (Invitrogen) to commercial RNA-seq analysis (Wuhan Aiji Baike Biotechnology Co., Ltd., Wuhan, China). The libraries were prepared by MGIEasy RNA Library Prep Set (Cat. No.: 1000006384; MGI Tech Co., Ltd., Shenzhen, China) and sequenced by using an illumine Hiseq system (PE150) following the recommended protocol. The raw reads were filtered out though fastp software ( https://github.com/OpenGene/fastp). Clean reads were mapped to the reference genome of Homo sapiens GRCh38 by Hisat2 ( https://ccb.jhu.edu/software/hisat2). The mapped reads of each sample were assembled by StringTie ( https://ccb.jhu.edu/software/stringtie). A comprehensive transcriptome was reconstructed through Gffcompare ( https://github.com/gpertea/gffcompare). StringTie was used to perform the expression level for mRNAs by calculating FPKM. The significantly differential expressions were selected with log2 [fold change]<0.5 and with Benjamini-Hochberg corrected *P*-value<0.05.

### Tumorigenesis

For the xenograft tumor model, female BALB/c nude mice aged 4–6 weeks were purchased from Hunan SJA Laboratory Animal Co. (Changsha, China). The cells as well as the matrix gel mixture were injected directly into the mice’s inguinal mammary fat pad (n=5/group). The number of cells to be grown varies according to different cell lines. HCC1806 and MDA-MB-468 cell cultivation numbers were 1×10 ^5^ and 1×10 ^6^, respectively. Tumor volumes, tumor weights, and body weights were monitored every 3 days until the end of the experimental period. Finally, tumor tissues were collected for the subsequent RT-PCR and IHC assays. Animal experiments were approved by the Animal Ethics Committee of the Kunming Institute of Zoology, Chinese Academy of Sciences (Kunming, China).

### Immunohistochemistry assay

The xenografted tumor tissues were fixed with 4% paraformaldehyde solution and dehydrated, and immunohistochemical assays were performed on approximately 4-μm-thick paraffin sections with antigen repair following pressure boiling. After incubation with the appropriate primary and secondary antibodies. Visualization of the signal on the slides was carried out by DAB staining. Ten fields of each group of samples were captured randomly using a microscope (Olympus, Tokyo, Japan), and the number of positive expressions of Ki67 and Cyclin D1 were calculated and averaged for quantification.

### Statistical analysis

Data were analyzed using GraphPad Prism 6 (GraphPad Software, San Diego, USA), and all data are expressed as the mean±SD or the mean±SEM as indicated. Student’s *t* test and two-way ANOVA were used to make comparisons between two and multiple groups, respectively. A *P* value<0.05 was regarded as statistically significant.

## Results

### ANG1 is highly expressed in TNBC and associated with poor prognosis

**Figure 1:**
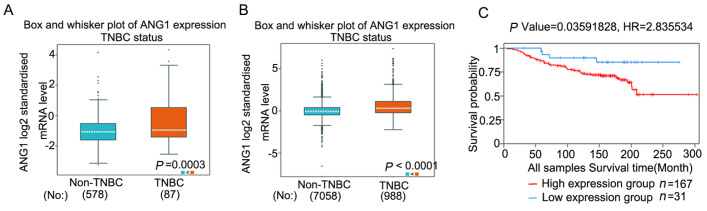
Figure 1 ANG1 is highly expressed in TNBC and associated with poor prognosis (A,B) ANG1 mRNA expression levels in TNBC and non-TNBC clinical samples were analyzed using bc-GenExMiner 4.5. (C) The TCGA database showed that high ANG1 expression in triple-negative breast cancer had a poor prognosis by BCIP.

ANG1 plays a critical role in solid tumor angiogenesis, but there are limited reports in its effects on TNBC. Exploration of various databases of clinical samples shows that *ANG1* mRNA levels are higher in TNBC than in non-TNBC ( Figure 1A,B). The relationship between ANG1 and survival in TNBC was analyzed with the TCGA database. High *ANG1* expression was associated with shorter overall survival (OS) ( Figure 1C).

### ANG1 promotes the proliferation of TNBC cells

**Figure 2:**
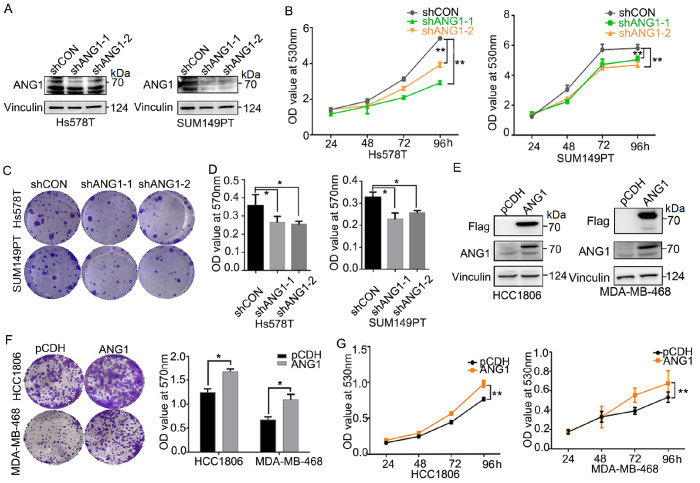
Figure 2 ANG1 promotes the proliferation of TNBC cells (A) Downregulation of ANG1 protein level was detected by western blot analysis. (B) Knockdown of ANG1 decreased the proliferation of Hs578T and SUM149PT cells, as detected by the SRB assay. Data are shown as the mean±SD. **P<0.01, two-way ANOVA. (C,D) ANG1 knockdown inhibited the colony formation of Hs578T and SUM149PT cells, as detected by colony formation assays. *P<0.05, t test. (E) Ectopic expression of ANG1 in HCC1806 and MDA-MB-468 cells was detected by western blot analysis. (F,G) Overexpression of ANG1 promotes the proliferation of HCC1806 and MDA-MB-468 cells, as detected by colony formation assays and SRB, respectively. Data are shown as the mean±SD. *P<0.05, **P< 0.01, t test and two-way ANOVA.

To investigate the effect of ANG1 on TNBC, we detected ANG1 expression in TNBC cell lines ( Supplementary Figure S1A) and chose four cell lines for further study. We knocked down *ANG1* in Hs578T and SUM149PT cells, in which the ANG1 protein levels were high ( Figure 2A and Supplementary Figure S1B), and overexpressed ANG1 in HCC1806 and MDA-MB-468 cells, in which the ANG1 protein levels were low ( Figure 2E). The results of SRB and colony formation assays indicated that *ANG1* knockdown significantly suppressed the proliferation of Hs578T and SUM149PT cells ( Figure 2B–D and Supplementary Figure S1C), and ANG1 overexpression significantly increased the proliferation of HCC1806 and MDA-MB-468 cells ( Figure 2F,G).

Glycosylated ANG1 is a secretory protein. To determine whether the effect of ANG1 on cell proliferation was based on its secretion, we collected conditioned medium (CM) from HCC1806 and MDA-MB-468 cells ectopically overexpressing ANG1 ( Supplementary Figure S1D,E) to culture HCC1806 and MDA-MB-468 cells, respectively. As a result, TNBC cells overexpressing ANG1 proliferated faster than their control cells ( Figure 2G), but the CM from ANG1-overexpressing cells had no effect on the proliferation of HCC1806 and MDA-MB-468 cells ( Supplementary Figure S1F). These results suggested that the pro-proliferation effect of ANG1 on TNBC cells does not occur in an autocrine manner.

### *ANG1* knockdown induces cell cycle G1 phase arrest and apoptosis

**Figure 3:**
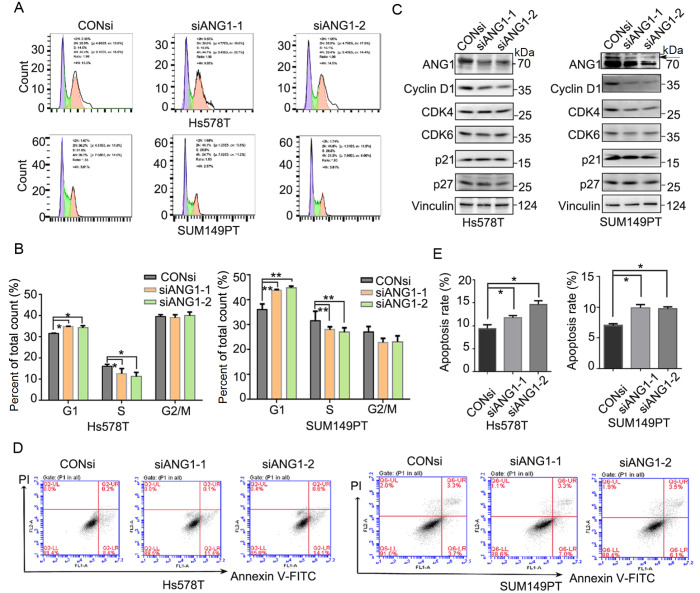
Figure 3 *ANG1* knockdown induces cell cycle G1 phase arrest and apoptosis (A) Hs578T and SUM149PT cells were treated with ANG1 siRNA (50 nM) for 48 h. Cells were stained with PI and analyzed by flow cytometry. The cell cycle graph was analyzed by FlowJo software. (B) ANG1 knockdown significantly increased the percentage of G1 phase cells compared to the control siRNA group. Data are shown as the mean±SD. *P<0.05, **P<0.01, t test. (C) After treatment with ANG1 siRNA (50 nM) for 48 h, cell lysates were collected for western blot analysis to detect Cyclin D1, CDK4, CDK6, p21, and p27 protein levels. Vinculin was used as a loading control. (D) Hs578T and SUM149PT cells were treated with ANG1 siRNA (50 nM) for 48 h. Cells were stained with Annexin V/PI and analyzed by flow cytometry. (E) Quantitative data of the percentage of Annexin V-positive cells. Data are shown as the mean±SD. *P<0.05, t test.

From a mechanistic perspective, we examined the effect of ANG1 on the cell cycle by flow cytometry. In Hs578T and SUM149PT cells, knockdown of *ANG1* increased the proportion of G1 phase but reduced the proportion of S phase, which suggested that *ANG1* knockdown decreased the G1-S transition ( Figure 3A,B). Consistent with this finding, downregulation of ANG1 expression diminished the expression of Cyclin D1, a cell cycle protein associated with the G1/S switch. There were no uniform changes in the expressions of CDK4, CDK6, p21 and p27 in Hs578T and SUM149PT cells ( Figure 3C). We subsequently investigated the effect of *ANG1* knockdown on apoptosis by flow cytometry. The results indicated that *ANG1* knockdown increased the percentage of apoptotic cells in both Hs578T and SUM149PT cell lines ( Figure 3D,E). In conclusion, these results suggest that ANG1 promotes TNBC cell proliferation and survival by regulating cell cycle progression and apoptosis.

### CPA4 is a downstream molecule of ANG1 in TNBC cells

**Figure 4:**
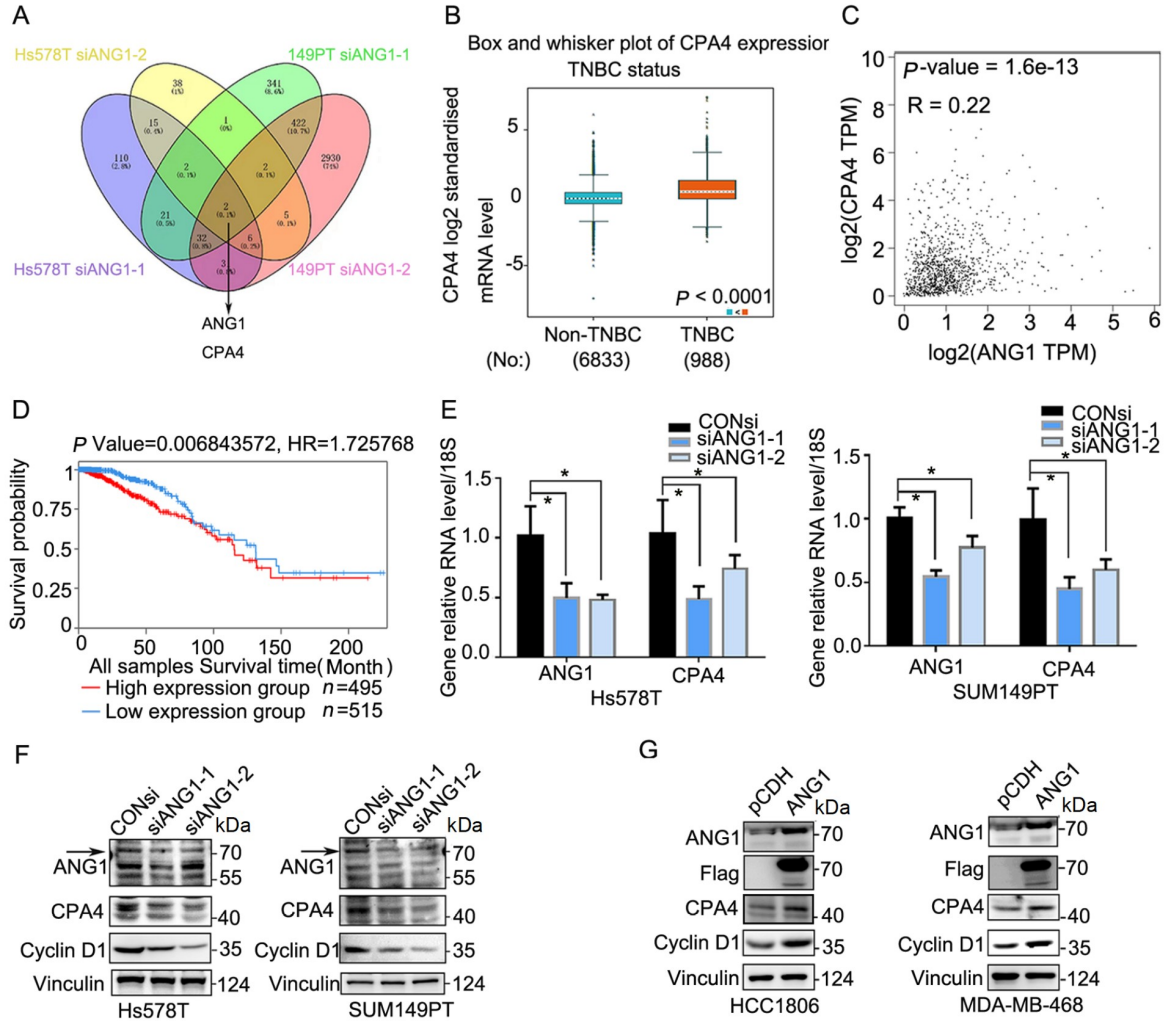
Figure 4 CPA4 is a downstream molecule of ANG1 in TNBC cells (A) RNA-seq was used to analyze the downstream regulatory molecules that knockdown ANG1 in Hs578T and SUM149PT cells. (B) CPA4 mRNA expression levels in TNBC and non-TNBC clinical samples were analyzed using bc-GenExMiner 4.5. (C) GEPIA was used to analyze Spearman’s correlation between ANG1 and CPA4 in breast cancer patients. (D) Overall survival analysis of TNBC patients with high CPA4 expression by TCGA database. (E) Real-time PCR was used to detect CPA4 mRNA levels after ANG1 knockdown. Data are shown as the mean±SD. * P<0.05, t test. (F) After treatment with ANG1 siRNA (50 nM) for 48 h in Hs578T and SUM149PT cells. Western blot analysis was used to detect CPA4 and Cyclin D1 protein levels. Vinculin was used as a loading control. (G) Cell lysates of ANG1-overexpressing cells were collected, and the protein levels of CPA4 and Cyclin D1 were detected by western blot analysis. Vinculin was used as a loading control.

To identify the specific mechanism by which ANG1 promotes TNBC, we knocked down *ANG1* in Hs578T and SUM149PT cell lines by using siRNA and performed RNA sequencing. By analyzing the RNA sequencing data, we identified carboxypeptidase A4 ( *CPA4*) as a downstream target gene of ANG1 ( Figure 4A). In clinical breast cancer samples, site analysis showed that CPA4 expression in TNBC was higher than that in non-TNBC ( Figure 4B), and there was a positive correlation between CPA4 and ANG1 in breast cancer ( Figure 4C). Overall survival analysis indicated that higher expression of CPA4 was prognostic for poorer overall survival ( Figure 4D). Western blot analysis was carried out to validate the relationship between CPA4 and ANG1. The results showed that *ANG1* knockdown reduced the protein levels of CPA4 and Cyclin D1 ( Figure 4F). qPCR showed that the *CPA4* mRNA level was also decreased in response to the reduction in ANG1 ( Figure 4E). In addition, ANG1 overexpression significantly increased CPA4 and Cyclin D1 protein levels ( Figure 4G). In conclusion, these findings suggest that CPA4 is a downstream molecule of ANG1 in TNBC.

### ANG1 promotes the proliferation of TNBC cells by upregulating CPA4

**Figure 5:**
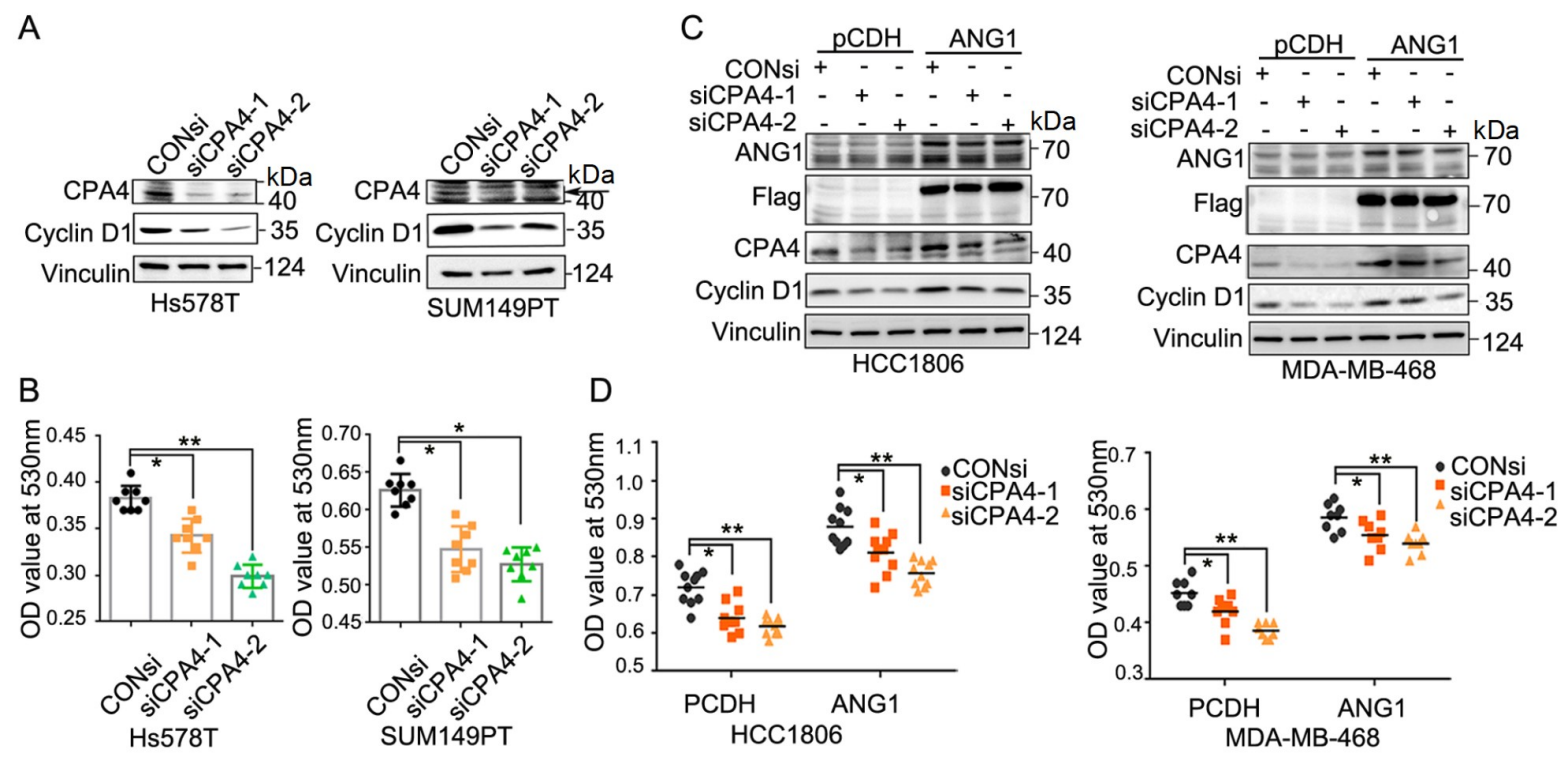
Figure 5 ANG1 promotes the proliferation of TNBC cells by upregulating CPA4 (A) After treatment with CPA4 siRNA (50 nM) for 48 h in Hs578T and SUM149PT cells. Western blot analysis was performed to detect Cyclin D1 protein level. Vinculin was used as a loading control. (B) Depletion of CPA4 expression suppressed Hs578T and SUM149PT cell proliferation. The cells were transfected with CPA4 siRNAs, and cell viability was measured by the SRB assay. Data are shown as the mean±SD. * P<0.05, **P<0.01, t test. (C) Stable ANG1-overexpressing cells were obtained by lentivirus infection and puromycin screening. Cell lysates were collected after treatment with CPA4 siRNA (50 nM) for 48 h, and CPA4 and Cyclin D1 protein levels were detected by western blot analysis. (D) CPA4 knockdown partially rescued the effect of ANG1 overexpression on cell proliferation. Data are shown as the mean±SD. *P<0.05, **P<0.01, t test.

To further investigate whether CPA4, as a downstream regulator of ANG1, has a similar function as ANG1, we knocked down *CPA4* by using siRNAs in Hs578T and SUM149PT cell lines and performed western blot analysis and SRB assay. The results revealed that *CPA4* knockdown resulted in substantial downregulation of Cyclin D1 ( Figure 5A) and inhibition of TNBC cell proliferation ( Figure 5B). Furthermore, *CPA4* knockdown partially blocked the increase in Cyclin D1 ( Figure 5C) and cell proliferation ( Figure 5D) induced by ANG1 overexpression. These findings suggested that ANG1 promotes TNBC through its downstream target gene CPA4, in part.

### ANG1 promotes tumor growth in TNBC xenograft models

**Figure 6:**
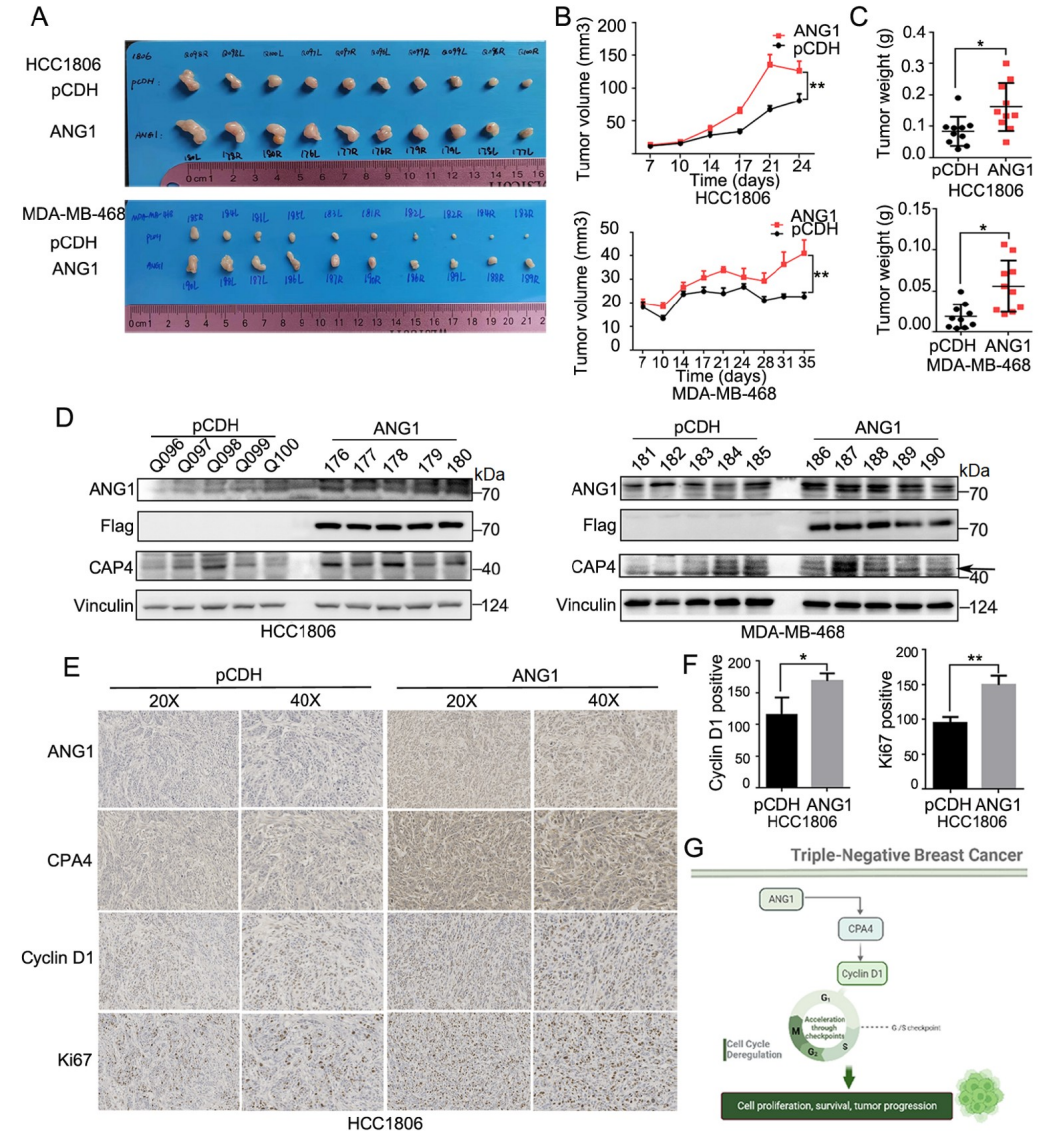
Figure 6 ANG1 promotes tumor growth in TNBC xenograft models (A–C) Representative images of the xenograft tumors isolated from the two indicated groups at approximately week 4. The tumor volumes of xenograft tumors isolated from the pCDH and ANG1 overexpression groups were analyzed by dot plot. Data are shown as the mean±SEM. n = 5. *P<0.05, ** P<0.01, t test. (D) Western blot analysis was used to detect the expression of CPA4 in pCDH and ANG1-overexpressing tissues (HCC1806 and MDA-MB-468). (E,F) IHC was used to analyze the expressions of the proliferation index Ki67, CPA4 and Cyclin D1 in xenograft tumors from mouse models. Data are shown as the mean±SD. *P<0.5, ** P<0.01, t test. (G) A schematic model of ANG1 playing a cancer-promoting role by upregulating CPA4 and Cyclin D1 using BioRender.

To characterize whether ANG1 promotes tumor growth *in vivo*, HCC1806 and MDA-MB-468 cells with stable ANG1 overexpression were injected into the fourth pair of mammary fat pads of 6-week-old female nude mice. Tumor volume and weight were both obviously higher in the ANG1 stable overexpression group than in the control group ( Figure 6A–C), and the mouse body weights were similar between the two groups ( Supplementary Figure S2A). Protein samples from tumor tissues showed that CPA4 level was also increased in the ANG1 overexpression group in comparison with the control group ( Figure 6D). The mRNA level of *CPA4* in tissues overexpressing ANG1 was upregulated ( Supplementary Figure S2B). In addition, the expressions of CPA4, Cyclin D1 and the proliferation indicator Ki67 were significantly higher in the ANG1 overexpression group than those in the control group, as revealed by IHC analysis ( Figure 6E,F and Supplementary Figure S2C,D). Collectively, these data suggest that ANG1 promotes tumor growth in TNBC.

## Discussion

In this study, we demonstrated that ANG1 promotes the proliferation and survival of TNBC by upregulating CPA4 ( Figure 6G). Mechanistically, ANG1 was shown to increase Cyclin D1 expression and G1/S cell cycle progression as well as inhibit apoptosis. These findings revealed that the ANG1-CPA4 axis may provide new therapeutic targets in TNBC.

The perivascular cells of mature blood vessels express ANG1, which plays an essential role in the development and stabilization of blood vessels [ 32, 33], and ANG1 has more recently been reported to be expressed in various tumor cells, such as breast cancer, gastric cancer and hepatocellular carcinoma. Accumulating evidence suggests that ANG1 is associated with tumor progression [ 34– 36] and poor survival [37] because it exhibits distinct correlations with cancer-associated fibroblasts [ 38, 39], endothelial cells [40], and the tumor microenvironment [ 34, 41, 42]. ANG1 was reported to be a glycosylated secretory protein that influences endothelial cell proliferation, migration and tube formation in a paracrine manner [15]. Interestingly, our study found that ANG1 promotes the proliferation of TNBC cells in a non-secretory manner.

CPA4 acts as a pro-oncogenic element in various tumors and is capable of promoting tumor progression [ 19, 43], EMT [17], metastasis and stemness [30]. *CPA4* knockdown has been reported to inhibit lung cancer cell proliferation by inducing cell cycle G1 arrest, downregulation of Cyclin D1 and apoptosis. For the first time, we showed that ANG1 positively regulates CPA4 at the mRNA and protein levels and promotes TNBC cell proliferation by upregulating the CPA4/Cyclin D1 axis. However, how ANG1 promotes the transcription of CPA4 was not explored, which is valuable for us to further investigate. In addition, the influence of the ANG1-CPA4 regulatory axis on other cellular functions, including stemness, metastasis, and drug resistance, requires further study.

In conclusion, we identified a novel mechanism by which ANG1 promotes TNBC proliferation and progression in a nonvascular regulatory manner, including pro-survival and antiapoptotic effects, especially the positive regulation of CPA4 by ANG1. Moreover, our results suggest that ANG1-CPA4 is a promising new direction for the treatment of TNBC patients and exploration of effective medications for clinical application.

## Supporting information

049supplementary_data_upload

## Supplementary Data

Supplementary data is available at *Acta Biochimica et Biophysica Sinica* online.
