# Supplementary material for: Angiopoietin-1 promotes triple-negative breast cancer cell proliferation by upregulating carboxypeptidase A4: ANG1-CPA4 axis promotes TNBC progression
Source: Acta Biochim Biophys Sin (Shanghai). 2023 May 9;55(9):1487–95. doi: 10.3724/abbs.2023082 (PMC10520468; doi:10.3724/abbs.2023082)
Supplement: 049supplementary_data_upload [file 049supplementary_data_upload.pdf]

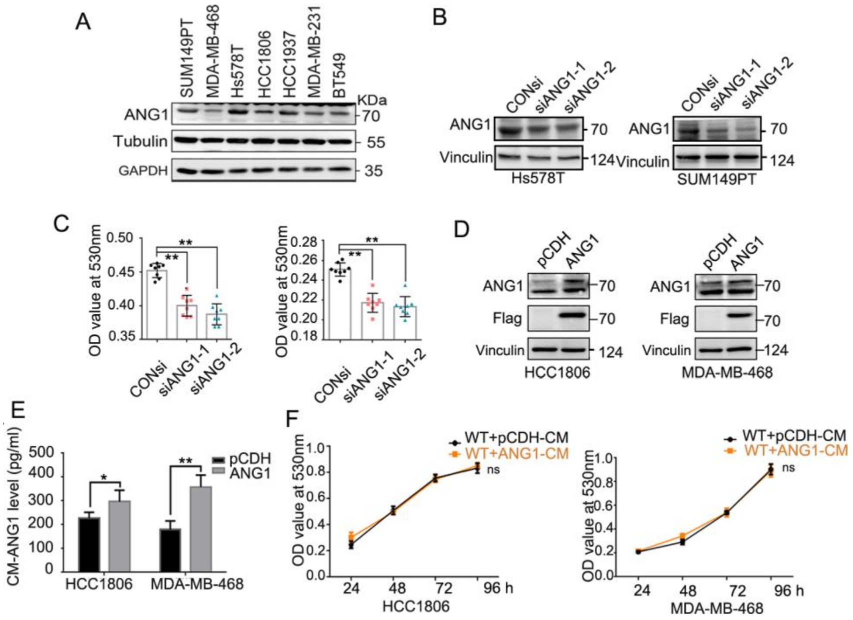

Supplementary Figure S1. ANG1 promotes the proliferation of TNBC cells (A) Western blot analysis was used to detect ANG1 protein expression in TNBC cell lines. (B) Downregulation of ANG1 protein levels in Hs578T and SUM149PT were detected by western blot analysis. (C) Knockdown of *ANG1* decreased the proliferation of Hs578T and SUM149PT cells, as detected by the SRB assay. $^{**}P < 0.01$, t test. (D) Ectopic expression of ANG1 in HCC1806 and MDA-MB-468 cells was detected by western blot analysis. (E) Conditioned medium (CM) from HCC1806 and MDA-MB-468 cells ectopically overexpressing ANG1 were collected to detect ANG1 level in CM. $^{*}P < 0.05$, $^{**}P < 0.01$, t test. (F) The CM from ANG1-overexpressing cells had no effect on the proliferation of HCC1806 and MDA-MB-468 cells. two-way ANOVA.

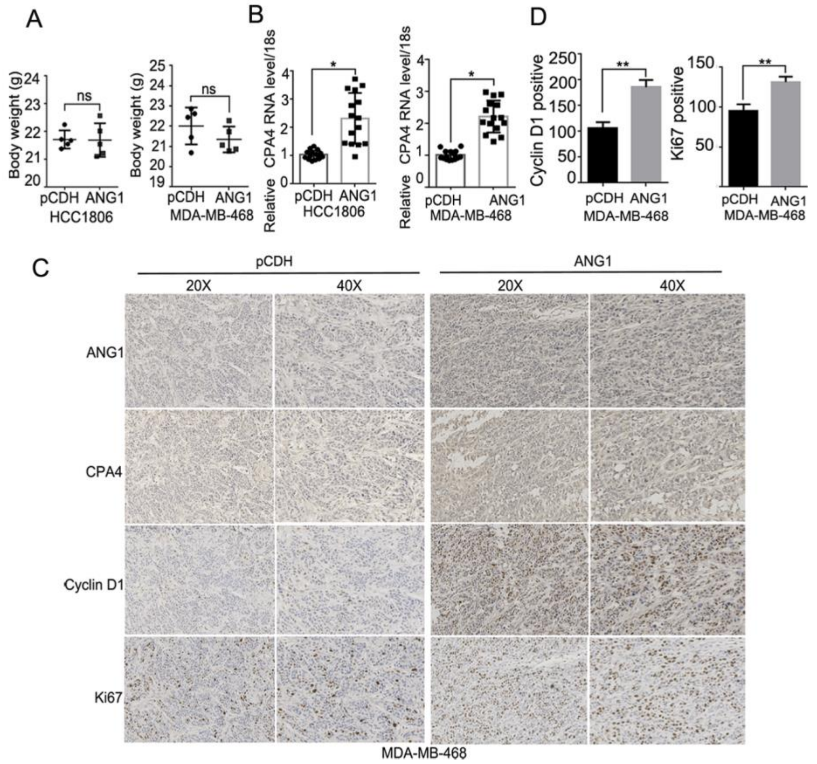

Supplementary Figure S2. ANG1 promotes tumor growth in TNBC xenograft models (A) No significant change in body weight of mice in the overexpression and control groups. (B) RT-PCR was used to detect the mRNA levels of CPA4 in pCDH- and ANG1-overexpressing tissues (HCC1806 and MDA-MB-468). **P* < 0.01, *t* test. (C, D) IHC was used to analyze the expressions of the proliferation index Ki67, CPA4 and Cyclin D1 in xenograft tumors from mouse models. Data are shown as the mean ± SD. **P* < 0.5, ***P* < 0.01, *t* test.
